# Wearable Electrochemical Biosensors for Monitoring and Management of Chronic Wounds

**DOI:** 10.3390/bios15120785

**Published:** 2025-12-01

**Authors:** Lingxia Zuo, Yinbing Liu, Jianrong Zhang, Linlin Wang, Jun-Jie Zhu

**Affiliations:** 1 School of Basic Medical Sciences, Ningxia Medical University, Yinchuan 750004, China; zuolingxia2017@nxmu.edu.cn; 2 State Key Laboratory of Analytical Chemistry for Life Science, School of Chemistry and Chemical Engineering, Nanjing University, Nanjing 210023, China; 602024240077@smail.nju.edu.cn (Y.L.); jrzhang@nju.edu.cn (J.Z.); 3 Shaanxi Key Laboratory of Chemical Additives for Industry, College of Chemistry and Chemical Engineering, Shaanxi University of Science and Technology, Xi’an 710021, China

**Keywords:** wearable electrochemical biosensor, chronic wound monitoring, infection diagnosis, real-time sensing, closed-loop theranostics, intelligent wound management

## Abstract

Chronic wounds constitute a major global public health challenge, characterized by a high risk of infection, prolonged healing times, and frequent recurrence. Conventional wound assessment methods, which primarily rely on visual clinical inspection and laboratory-based analyses, are limited by inherent subjectivity, delayed feedback, and a lack of capacity for real-time monitoring of the dynamic biochemical changes at the wound site. Significantly, recent advancements in flexible electronics, nanomaterials, and energy harvesting technologies have boosted the rapid development of wearable electrochemical biosensors. These devices have emerged as a transformative platform for the continuous, non-invasive analysis of critical biomarkers within the wound microenvironment, including pH, temperature, inflammatory cytokines, metabolites, and pathogen-derived molecules. This review critically examines the latest progress in wearable electrochemical biosensors for wound monitoring and management. Key discussions include (1) the special requirements for sensor design, targeting the chronic wound’s pathological characteristics; (2) cutting-edge development in self-powered systems, multimodal sensor integration, closed-loop theranostics, and artificial intelligence (AI)-assisted decision-making; and (3) a critical appraisal of challenges in accuracy, stability, biocompatibility, energy management, and clinical translation. Finally, the review explores future trends, such as biodegradable sensors, multi-parameter fusion algorithms, and remote intelligent management systems, with the aim of establishing a foundational framework and providing technical guidance for developing next-generation intelligent wound care solutions.

## 1. Introduction

Chronic wounds represent a significant challenge for global healthcare systems. Characterized by prolonged healing cycles, high infection risk, and high recurrence rates, chronic wounds significantly diminish patients’ quality of life while simultaneously imposing a substantial economic burden on healthcare systems [1,2]. Epidemiological data firmly establish a critical link between infection and adverse outcomes in chronic wounds, for instance, moderate-to-severe infections in diabetic foot ulcers (DFUs) precede amputations in approximately 20% of cases. Traditional treatments focus on debridement, moisture-retentive dressings, and antibiotic therapy [3]. However, chronic wound pathogenesis is highly complex, involving factors such as vascular insufficiency, neuropathy, dysregulated immune responses, and persistent bacterial infection. And the healing process is frequently impeded by abnormal fluctuations in key biochemical and biophysical markers within the wound microenvironment (e.g., pH, temperature, enzyme activity, and inflammatory mediator levels) [4,5]. Therefore, the real-time and dynamic monitoring of these critical physiological parameters is vital for accurately assessing wound status and guiding personalized therapeutic strategies.

Current wound evaluation methods predominantly rely on subjective visual inspection and manual documentation technology, such as measuring wound area, evaluating exudate characteristics, assessing tissue coloration, and recording patient-reported pain levels. These approaches are inherently subjective, exhibit poor reproducibility, and are incapable of delivering quantitative molecular-level information. Diagnosis of wound infection and inflammation status often depends on time-consuming laboratory culture and blood tests, which inevitably result in significant delays in therapies [6,7,8]. Furthermore, the key physiological markers within the wound bed (e.g., pH, redox molecules, protease, inflammatory cytokines) undergo rapid and dynamic fluctuations throughout the healing trajectory [9,10,11]. Conventional intermittent clinical evaluations or laboratory tests are fundamentally inadequate for capturing these transient yet critical variations. Consequently, the existing conventional techniques fail to meet the urgent clinical need for high-frequency, real-time, and point-of-care wound monitoring, a critical limitation given the extended management cycles and narrow therapeutic windows associated with chronic wound management.

Biosensors are analytical devices that transduce specific biological or chemical reactions into quantifiable electrical or optical signals [12,13]. Recent advances in materials science, micro/nanofabrication, and flexible electronics have accelerated the application of biosensors for chronic wound management. In contrast to conventional methods, biosensors display potential for the non-invasive, continuous and real-time monitoring of key biomarkers within the wound microenvironment (e.g., inflammatory cytokines, bacterial metabolites, oxidative stress markers), with high sensitivity and specificity [14,15]. The integration of these sensors into wound dressings to construct “intelligent wound dressing platforms” has the potential to revolutionize wound care. Such platforms not only facilitate the real-time tracking of healing progress and infection risk but can also be coupled with closed-loop therapeutic systems to enable on-demand drug release or microenvironment modulation. This can provide an integrated “monitoring-response-treatment” paradigm for intelligent wound management [16,17]. Furthermore, the ongoing advances in the miniaturized biosensors have yielded wearable sensing devices that are compatible with home-based care and telemedicine, thereby reducing the need for hospitalization, lowering the healthcare cost, and offering a new pathway for managing chronic wounds.

Among biosensing technologies, electrochemical biosensors have gained prominence as a leading platform for chronic wound monitoring due to their high sensitivity, rapid response, miniaturization potential, and compatibility with system integration [18]. Biological electrical signals play a crucial role in maintaining human physiological homeostasis, including the conduction of neural action potentials, regulation of tissue-level internal environmental balance, and the re-epithelialization process in skin wounds [19]. Recent studies have demonstrated that electrochemically driven targeted electric field interventions can effectively modulate the wound microenvironment. By activating voltage-gated ion channels and intracellular signaling pathways, these interventions synergistically promote cell migration, angiogenesis, and anti-inflammatory responses. Moreover, electrochemical biosensors harness biochemical energy, such as glucose/O_2_, from tissue fluid in situ and directly convert it into therapeutic electrical signals. These systems establish a continuous and controllable electric field at the wound edge (<10 mV/mm) while simultaneously enabling real-time monitoring of dynamic biomarker changes during the healing process [20]. By translating variations in biomarker concentration into measurable electrical signals, including current, voltage, or impedance, these sensors achieve high-frequency and dynamic tracking of wound status [21,22]. Additionally, electrochemical sensors display high scalability and excellent compatibility with advanced functional materials, including conductive polymers, nanocarbon materials and metal nanoparticles, which can be engineered to enhance both sensitivity and selectivity toward specific biomarkers [23]. Furthermore, their simple structure, low fabrication cost, and minimal power consumption enable them to be easily integrated into flexible substrates, microneedle patches, or portable wireless systems, thereby promoting the transition toward intelligent and personalized wound care paradigms.

The realization of continuous and accurate monitoring using wearable electrochemical sensors critically depends on the development of sustainable and stable power sources. Recent advances in wearable energy harvesting technologies present a viable pathway to address this limitation. Such systems are capable of harvesting energy from various biological sources (e.g., biomechanical, biochemical, or thermal origins) or the ambient environment (e.g., solar). In certain designs, these energy harvesters can also function as active sensing elements, directly detecting physical deformations or specific biochemical cues. The integration of energy harvesting modules with electrochemical sensing platforms paves the way for self-powered, long-term intelligent monitoring systems, thereby facilitating truly wearable, battery-free solutions ideally suitable for chronic wound management [24,25,26].

In summary, wearable electrochemical biosensors represent a paradigm shift in chronic wound management. Through the integration of advances in flexible electronics, micro/nano-sensing, energy harvesting, and intelligent algorithms, the wearable electrochemical biosensors enable in situ, continuous, dynamic monitoring of key physiological parameters within the wound microenvironment. This capability provides unprecedented data support for early infection warning, healing status assessment, and therapeutic strategy optimization. The impressive advances in self-powered technologies, closed-loop theranostic systems, and AI-assisted analysis, are actively advancing chronic wound care from a traditional reactive paradigm toward proactive intervention and personalized regulation. However, the clinical translation and widespread adoption still face significant challenges, including achieving stable and sustainable power sources, maintaining sensing reliability in complex biofluids, mitigating signal crosstalk in multiplexed detection, as well as overcoming manufacturing and cost-related barriers. Future efforts should focus on the development of novel functional materials, innovative device architecture, system-level energy optimization, and extensive clinical trials. Ultimately, deep integration across engineering, medical, and data science will be essential to build truly reliable, safe, and practical intelligent wound management systems that can improve patient outcomes and alleviate healthcare burdens.

## 2. Pathophysiological Mechanisms of Chronic Wounds and Implications for Biosensor Design

### 2.1. Definition of Chronic Wound

Chronic wounds are clinically defined as skin injuries that fail to heal through the orderly physiological reparative processes, typically persisting beyond four weeks despite appropriate treatment. Prototypical cases include DFUs and pressure injuries [27,28,29]. These wounds typically originate from compromised skin integrity due to physical trauma, burns, persistent mechanical stress, or underlying pathologies such as diabetes and vascular insufficiency. Once wound management is inadequate, the resulting dysregulated and prolonged repair process will impose a severe health burden on patients, increasing risk of severe complications, such as amputation and mortality, alongside significant socioeconomic costs.

### 2.2. Pathophysiological Characteristics of Chronic Wound

**Figure 1:**
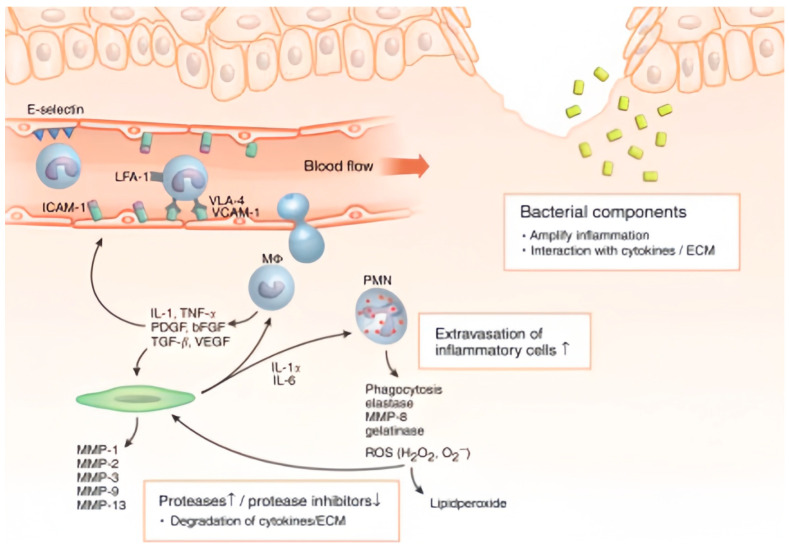
Model of multifactorial molecular and cellular mechanisms deleterious in tissue repair (↑ and ↓ represent an increase or decrease in content) [30]. Copyright 2007 Elsevier.

The healing of chronic wounds is impeded by a complex interplay of pathological mechanisms that disrupt the normal repair process. The core challenges include persistent inflammation, recurrent infection, tissue necrosis, and compromised re-epithelialization. Underlying systemic conditions and local stimuli may prolong the inflammatory phase of the local healing response, thereby triggering a cascade of tissue-level reactions that generate and sustain a hostile wound microenvironment (Figure 1) [30]. Chronic wounds fail to progress through the typical stages of wound repair, and instead remain trapped in a state of chronic inflammation, predominantly characterized by extensive infiltration of polymorphonuclear neutrophils (PMNs) and macrophages (Mɸs). Sustained activation of these inflammatory cells leads to the overproduction of pro-inflammatory cytokines, such as IL-1, TNF-α, and IL-6, and contributes to a protease-rich and pro-oxidant hostile microenvironment. Elevated proteolytic activity, mediated by neutrophil elastase, MMP-8, and gelatinase, results in the degradation of growth factors and structural extracellular matrix proteins crucial for tissue repair [31,32]. Increased level of ROS (H_2_O_2_, O_2_^−^) can directly damage cells and extracellular matrix molecules, or further upregulate the expression of MMPs (MMP-1, -2, -3, -9, and 13) [33]. Additionally, bacterial components, such as extracellular adherence protein (Eap), formyl methionyl peptides, and N-acetylmuramyl-L-alanyl-D-isoglutamine may impair host repair mechanisms by disrupting cell-matrix interactions or exacerbating the inflammatory response [34].

**Figure 2:**
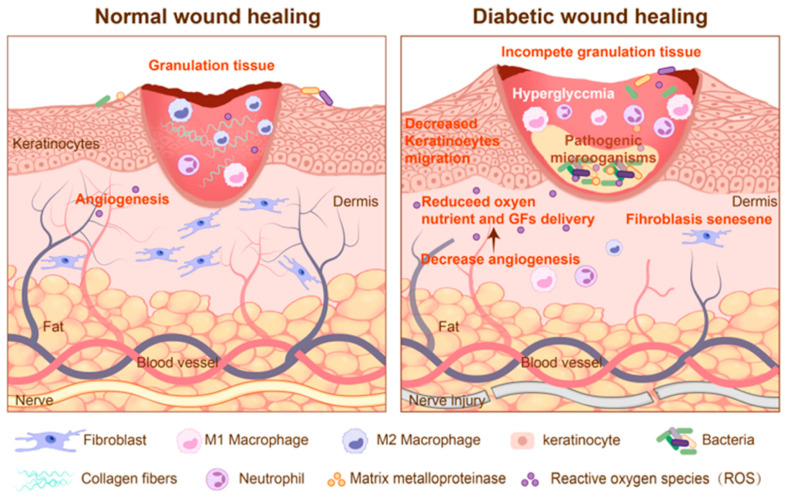
The physiological processes of normal wounds and diabetic wounds [41]. © 2025 by the authors.

Diabetic wounds are a typical type of chronic wounds. In diabetic wounds, hyperglycemia-induced pathological mechanisms disrupt repair processes at multiple levels. Chronically elevated glucose levels increase cellular oxygen consumption, leading to localized hypoxia and a surge in reactive oxygen species (ROS) [35]. Elevated ROS not only inflict direct damage on endothelial function but also activate signaling pathways such as ERK and EGFR, which upregulate pro-inflammatory factors like IL-8 to amplify neutrophil infiltration. Dysregulation of core morphogenic signaling pathways further impedes repair. Impaired TGF-β/SMAD signaling hinders the differentiation of fibroblasts into myofibroblasts, impeding wound contraction. Meanwhile dysregulated Wnt/β-catenin pathway disrupts stem cell mobilization and re-epithelialization [36]. In addition, hyperglycemia also promotes AGE accumulation. Binding of AGEs to their receptor (RAGE) not only disrupts ECM structure but also potently activates NF-κB, establishing a pro-inflammatory positive feedback loop [37]. Furthermore, cellular senescence exacerbates this dysfunction. Senescent cells secrete large amounts of inflammatory cytokines and proteases (e.g., IL-6, MMPs) via the senescence-associated secretory phenotype (SASP), which alter the microenvironment and impede normal cell function [38]. Finally, immune balance is significantly compromised, with failure of M1 macrophages to effectively polarize towards the reparative M2 phenotype, and defects in regulatory T cell (Treg) function impairing immune regulation [39,40]. Together, these factors create a hostile microenvironment that actively suppresses healing cascades in diabetic wounds. Compared to normal wounds, diabetic wounds exhibit dysregulated angiogenesis with chronic hypoxia, prolonged inflammatory responses, increased oxidative stress, persistent bacterial colonization, and concurrent neuropathy (Figure 2) [41].

### 2.3. Healing Impairment Factors and Sensing Targets

Factors impeding chronic wound healing include systemic and local determinants. Systemic factors include advanced age, underlying chronic diseases (e.g., diabetes, vascular insufficiency), and poor nutritional status. The local factors involve wound desiccation, infection, external pressure, and repeated trauma. These collectively impair cellular activities at the wound site [42]. Real-time, multi-parameter monitoring of these indicators holds promise for delivering tools to precisely assess wound status, guide debridement and pharmacotherapy, ultimately improving healing outcomes while alleviating the associated public health burden.

## 3. Recent Advances in Wearable Electrochemical Biosensors for Wound Management

Current research in wearable electrochemical biosensors is driving a paradigm shift, transforming these devices from simple detectors into sophisticated platforms central to intelligent wound management. This evolution hinges on three interlocking advancements: first, the development of advanced functional materials enabling sensitive, biocompatible, and durable on-body integration; second, the implementation of closed-loop management systems that achieve seamless real-time monitoring coupled with autonomous therapeutic intervention; and third, the synergistic combination of multi-modal sensing with AI-enhanced diagnostic analysis, unlocking unprecedented capabilities for complex wound microenvironment interpretation and adaptive decision-making. This section systematically reviews the forefront progress within these interconnected domains, establishing their collective role in realizing the vision of truly personalized, responsive, and effective wound care.

### 3.1. Development Status of Functional Materials

The electrode is the core component of the wearable biosensing systems, performing two key functions: (1) providing a stable platform for immobilizing biological receptor molecules; and (2) efficiently transducing biological recognition events into measurable electrical signals. The selection of electrode materials is critical, as it determines many performance parameters, particularly for surface-based applications, including but not limited to analytical sensitivity, measurement accuracy, signal stability, and operational durability.

Recent advances in electrode fabrication have incorporated diverse functional nanomaterials to enhance biosensor performance. These include metallic nanoparticles, carbon-based materials (e.g., carbon nanotubes and graphene), two-dimensional materials such as MXene), porous architectures (e.g., nanoporous gold and metal-organic frameworks), as well as conducting polymers [43,44,45]. When integrated into working electrodes, these nanomaterials provide three key advantages: (1) a significantly increased electroactive surface area that enhances signal generation, (2) enhanced electron transfer kinetics leading to shorter response times, and (3) greater bioreceptor loading capacity, thereby increasing detection sensitivity. While these material innovations have markedly improved the analytical capabilities of wearable sensing platforms, their clinical translation requires rigorous evaluation of biocompatibility. Particular attention must be paid to potential adverse effects, including foreign-body reactions and inflammatory responses, which necessitate rigorous preclinical testing and human validation prior to widespread implementation in wearable healthcare devices.

The selection of base materials also plays a fundamental role in the development of wearable biosensing platforms. Synthetic polymers and hydrogels have emerged as essential materials for device integration, fulfilling multiple critical functions including fluidic patterning, electrode encapsulation, and biological interfacing. Synthetic polymers, in particular, are widely used as flexible substrates for printed electronics in on-body applications due to their exceptional combination of mechanical flexibility, biocompatibility, and scalability in manufacturing. Commonly used elastomeric materials such as polydimethylsiloxane (PDMS), polyethylene terephthalate (PET), polyimide (PI), silicone rubbers, and poly(styrene-butadiene-styrene) copolymers offer distinct advantages for continuous monitoring, primarily due to their established skin compatibility and tunable mechanical properties (such as elasticity and stretchability) that can be engineered to match those of human tissues [46,47]. This mechanical compatibility ensures stable, conformal contact with biological surfaces during prolonged wear. Furthermore, the surface characteristics of these polymeric substrates can be precisely engineered through both physical treatments (e.g., oxygen plasma treatment to enhance hydrophilicity) and chemical functionalization approaches.

Hydrogel materials, such as poly (vinyl alcohol) (PVA), alginate, and gelatin formulations, represent another important class of substrate materials with unique advantages for biosensing applications [48]. These hydrophilic polymer networks demonstrate superior biocompatibility compared to synthetic elastomers, making them particularly suitable for direct interfacing with sensitive biological environments such as open wounds or mucosal surfaces [49,50]. Their inherent porosity and hydration capacity enable efficient biofluid permeation and transport, thereby improving sampling efficiency and reducing analytical delay. In practical implementations, hydrogels are frequently used as (1) functional matrices for enhanced fluid management, (2) antifouling coatings for electrodes, and (3) programmable reservoirs for drug delivery. However, broader adoption of hydrogels as primary substrate materials faces significant challenges, most notably the mechanical mismatch between the soft, compliant hydrogel matrix and the relatively rigid electrode components [51,52,53]. This disparity often leads to interfacial delamination and electrical discontinuity during extended operation in wet physiological environments. Additionally, the relatively high production costs of performance-optimized hydrogels currently restrict their use to specialized applications where their unique properties are indispensable.

### 3.2. Closed-Loop Theranostic Systems

**Figure 3:**
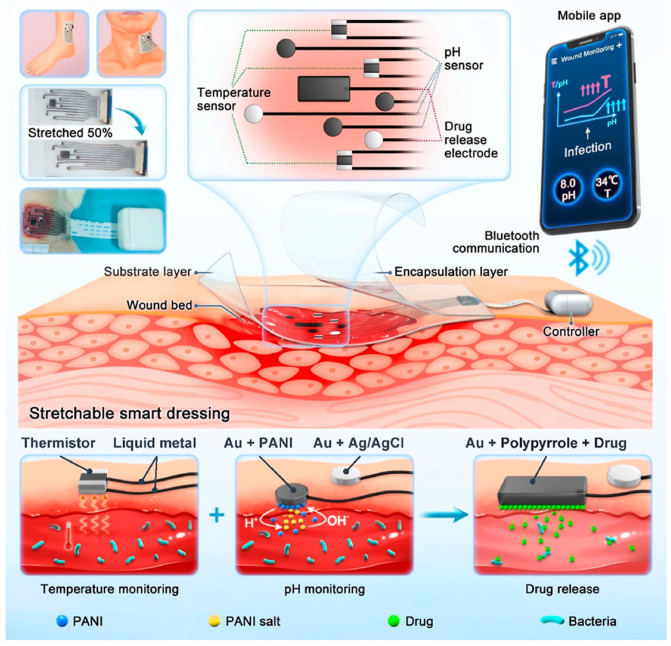
Soft stretchable smart wound dressing for wound infection monitoring and treatment. Schematic diagram of the smart dressing patched on the wound site for bacterial infection detection by simultaneous monitoring of the changes in temperature and pH value, and providing treatment via electrically controlled drug releasing (the red and blue arrows indicate the monitoring paths for temperature and pH value sensing signals) [56]. Copyright 2024 Elsevier.

Wearable electrochemical sensors are increasingly being integrated with therapeutic modules to form closed-loop theranostic systems, enabling intelligent wound management through cascaded “monitoring-feedback-regulation” functions. Recent advances have integrated various actuation modalities, such as controlled drug release systems, electrical stimulation, and microneedle-based delivery, to achieve closed-loop wound treatment [54,55]. Systems utilizing externally triggered electrical stimuli for controlled release have developed rapidly due to their efficacy in localized intervention. These systems typically feature multi-functional integration, wireless transmission, closed-loop control, user-friendly interfaces, flexibility, and portability, thereby enabling continuous monitoring and personalized therapy. By centering on electrochemical sensing, these systems enable the biomarker-controlled dynamic regulation of drug release, significantly improving therapeutic precision and adaptability. This integrated approach not only improves the intervention timeliness but also facilitates the development of self-powered, wearable medical devices for autonomous wound care. For instance, Su et al. [56] reported a stretchable dressing that integrated real-time temperature and pH sensing for wound status assessment with an electrochemically controlled drug delivery system (Figure 3). Specifically, the dressing is a dedicatedly designed three-layer patch, including an Ecoflex-based substrate, soft integrated circuit layer, and an ultra-thin encapsulation layer. The soft integrated circuit layer contains multiple sets of gold particle-based soft electrodes for dual-channel sensing of wound temperature and pH value, as well as an electrically controlled antibiotic-releasing system to combat wound infection. This device achieved stable conductive performance with a resistance change rate of less than 6% at a 50% strain through a liquid metal/gold composite electrode, thereby solving the mechanical mismatch issue between flexible electronics and soft tissues. Animal experiments demonstrated that the smart dressing was capable of detecting bacterial infection via the biomarkers of temperature (32.5 to 33.7 °C) and pH value (7.0–8.0) and the infection factors of wound were significantly improved with therapy through electrically controlled antibiotic releasing. However, achieving long-term, adaptive wound management necessitated not only function integration but also advances in material design. The next-generation platforms required enhanced biocompatibility and mechanical compliance, including stretchability and self-healing properties, to better conform to the dynamic wound environment and maintain stable operation over extended periods. In this regard, Zhang et al. [57] synthesized a multiple dynamically crosslinked PCPZ hydrogel (the PVA/B2/CS2/CS-PPY1 hydrogel was supplemented with 2% ZnCS), developed based on PVA and functionalized chitosan, exhibiting high stretchability (>3500%), autonomous self-healing capability, conductivity (1.16 S/cm), and strong antibacterial activity. This hydrogel was successfully utilized to fabricate a temperature sensor (linear response from 0 to 43.5 °C) and a strain sensor (linear strain-resistance relationship), which regained functionality rapidly after it was severed. In an established rat chronic wound model, PCPZ dressings combined with electrical stimulation significantly accelerated wound healing, manifested as faster closure rates, more complete tissue regeneration, and reduced inflammatory responses. This study provides an integrated material solution for flexible bioelectronics and smart wound dressings, offering dual sensing and therapeutic capabilities.

### 3.3. Multi-Modal Sensing and AI-Enhanced Diagnostic Analytics

**Figure 4:**
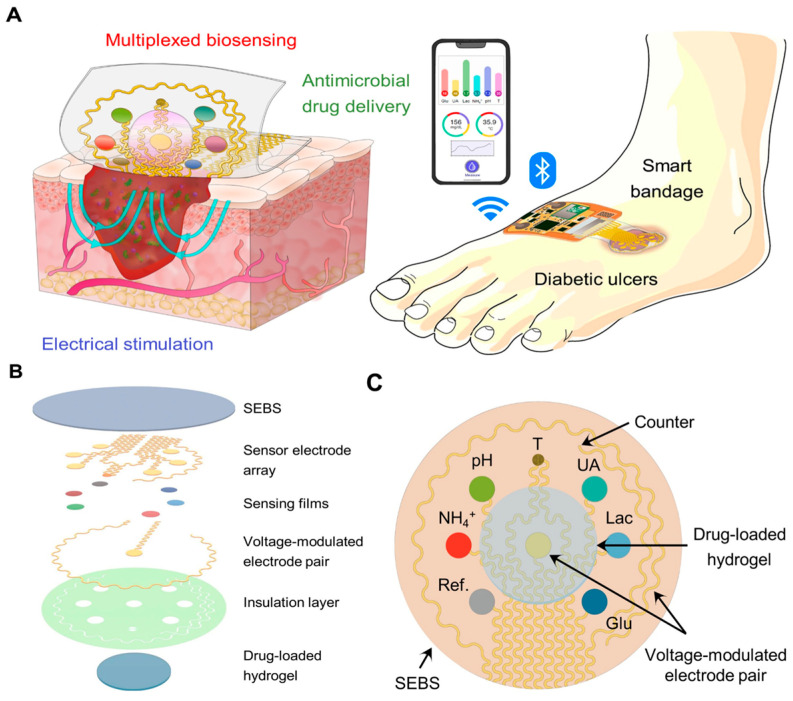
A wireless stretchable wearable bioelectronic system for multiplexed monitoring and treatment of chronic wounds. (**A**) Schematic of a soft wearable patch on an infected chronic nonhealing wound on a diabetic foot. (**B**) Schematic of layer assembly of the wearable patch, showing the soft and stretchable poly(styrene-b-(ethylene-co-butylene)-b-styrene) (SEBS) substrate, the custom-engineered electrochemical biosensor array, a pair of voltage-modulated electrodes for controlled drug release and electrical stimulation, and an anti-inflammatory and antimicrobial drug-loaded electroactive hydrogel layer r (the colors correspond to the functions as shown in Figure 4C). (**C**) Schematic layout of the smart patch consisting of a temperature (T) sensor, pH, ammonium (NH^4+^), glucose (Glu), lactate (Lac), and UA sensing electrodes, reference (Ref) and counter electrodes, and a pair of voltage-modulated electrodes for controlled drug release and electrical stimulation [61]. Copyright 2025 American Association for the Advancement of Science.

**Figure 5:**
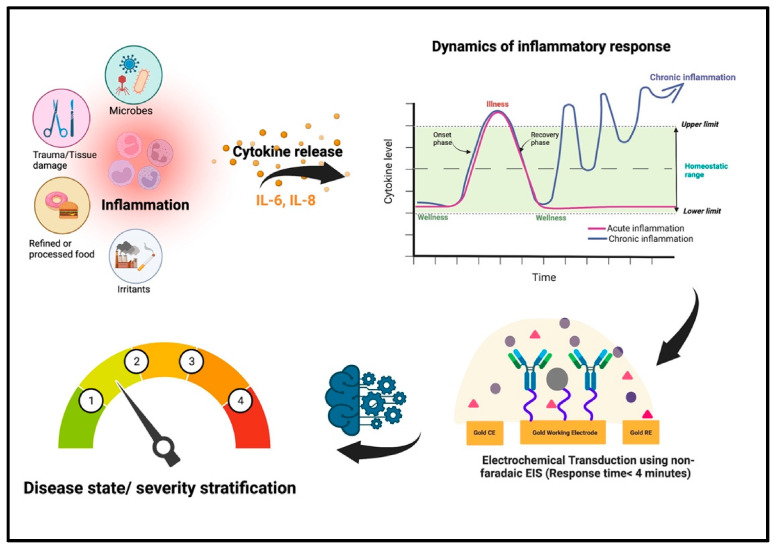
Schematic showing the operation of the proposed combinatorial inflammatory stimulus responsive biosensor (red triangles denote high biomarker concentrations; gray circles denote low biomarker concentrations) [62]. © 2022 by the authors.

Monitoring single biomarkers offers limited insight into the complex and dynamic wound microenvironment. Multi-target sensing systems, which combine multiple biochemical and physical sensing modalities, provide a more comprehensive data foundation for wound assessment [58,59,60]. These systems can simultaneously track chemical markers in wound exudate (e.g., IL-1β, IL-6, TNF-α, MMP-9) alongside key physiological parameters (e.g., pH, temperature), offering holistic insights. In one representative example, Shirzaei Sani et al. [61] developed a fully integrated, stretchable, wireless wearable bioelectronic system (WSBS, smart bandage) capable of synchronously detecting key wound biomarkers—temperature (25–45 °C), pH (7.0–9.0), glucose (0–40 mM), lactate (0–4 mM), uric acid (0–150 μM), and ammonium ions (up to 1 mM) (Figure 4). This platform successfully addresses critical clinical challenges in chronic wound management, including delayed healing, lack of real-time multi-parameter monitoring, and the limitations of single-modality therapies. Experimental results demonstrated that the system not only enables real-time tracking of dynamic biomarker changes across the wound infection-to-healing continuum, but also significantly accelerates wound healing in diabetic rat models through a combined therapeutic approach involving electrically controlled drug delivery and electrostimulation. This approach achieved 99% re-epithelialization, effectively reduced scar formation, and elucidated the underlying molecular mechanism of tissue regeneration. In a separate study, Ganguly et al. [62] developed a non-faradaic, EIS-based urinary inflammation biosensor capable of ultrasensitive detection of IL-6 and IL-8 (limit of detection (LOD) = 1 pg/mL) and four-state disease stratification. The sensor incorporates a flexible lateral-flow membrane integrated with a gold three-electrode system, enabling detection within less than four minutes while maintaining high selectivity against common interferents such as urea and glucose. By employing a two-stage random forest machine learning model, the system classifies four distinct physiological states—“Healthy,” “Pre-Inflammatory,” “Symptomatic,” and “Systemic Inflammation”—with an accuracy of 98.437%. This work represents the first demonstration of direct integration of raw impedance data into a machine learning model for multi-state disease classification, providing actionable and interpretable diagnostic outputs suitable for home-based point-of-care (POC) devices. This approach is particularly valuable for managing inflammatory conditions requiring rapid and accurate stratification (Figure 5).

The high-volume, multivariate data streams produced by multi-modal wearable sensors necessitate advanced analytical methods for meaningful interpretation. Artificial intelligence (AI), especially machine learning (ML), is pivotal for processing and interpreting complex data generated by wearable biosensors. Unlike conventional analysis methods, which often overlook the subtle patterns or correlations within high-dimensional datasets, ML technologies identify non-linear correlations, temporal trends, and early anomalies in real time. For instance, AI-based technologies can monitor changes in pH, temperature, and cytokine levels, predicting infection before overt clinical signs appear [63]. Deep learning models further enhance this capability by integrating multiple biomarker signals into a holistic view of the wound environment, enabling timely intervention to prevent complications and improve healing rates [64]. Additionally, AI supports personalized treatment by adapting system responses to individual patient physiology and evolving healing trajectories, thereby enabling dynamic treatment optimization. The convergence of AI with wearable biosensors drives a transformation in precision medicine, facilitating proactive, efficient, and patient-centered wound care. For example, Liu et al. [65] developed a bio-machine interface that integrated real-time multi-modal sensing with programmable therapeutic functions for critical wound care. The device—incorporating inert dermal matrix material—monitored glucose, temperature, pH, and humidity with extremely low energy consumption during stimulation events. Combined ML-assisted analysis enabled accurate wound assessment and closed-loop therapeutic feedback, thus accelerating healing, as evidenced by promoted dermal remodeling, neovascularization, and collagen deposition. The device also displayed antimicrobial efficacy against common pathogens. The system’s efficacy was rigorously validated through in vitro and in vivo studies, supported by transcriptomic profiling and microbiome analysis. This integrated platform combined biosensing and therapeutic functions within a single, clinically translational framework, demonstrating significant improvements in wound closure, attenuation of inflammation, and promotion of tissue regeneration. Leveraging AI technologies, clinicians can perform real-time wound status tracking and adaptive treatment management, enabling predictive analysis of healing trajectories. These strategies not only reduce the risk of transition from acute to chronic pathologies but also prevent exacerbation of refractory wounds through continuous optimization of the microenvironment. This approach significantly enhances diagnostic accuracy, therapeutic efficacy, and patient outcomes while facilitating early infection detection and streamlining clinical workflows.

## 4. Electrochemical Biosensors for Wound Monitoring and Healing

Wound healing is a highly dynamic and complex biological process involving meticulous coordination of numerous cellular and molecular events. This process is frequently impaired by factors such as bacterial infection, persistent inflammation, hyperglycemia, and elevated oxidative stress, often resulting in chronic nonhealing wounds [66,67,68]. Therefore, continuous, accurate real-time monitoring of wound status is essential for improving therapeutic outcomes and enabling personalized management.

### 4.1. Real-Time Wound Monitoring

Real-time monitoring aims to dynamically assess healing status and detect abnormalities by continuously tracking key biomarkers. Temperature is a vital indicator reflecting biochemical and cellular activities during tissue repair. Studies have indicated that normally healing wounds exhibit temperatures within a range of 31.1 °C to 36.5 °C, while deviations exceeding 2.2 °C strongly correlate with wound deterioration [69]. Subnormal temperatures are frequently related to altered circulation, reduced enzyme activity, or lymphopenia, whereas elevated temperatures indicate pathogenic infection or intense inflammation. The integration of highly sensitive and accurate temperature sensors—including infrared, colorimetric, and electrochemical sensors—into wound dressings offers considerable promise. Among these, flexible electronic sensors based on resistive or capacitive principles are particularly advantageous due to their conformability and precision for continuous monitoring.

**Figure 6:**
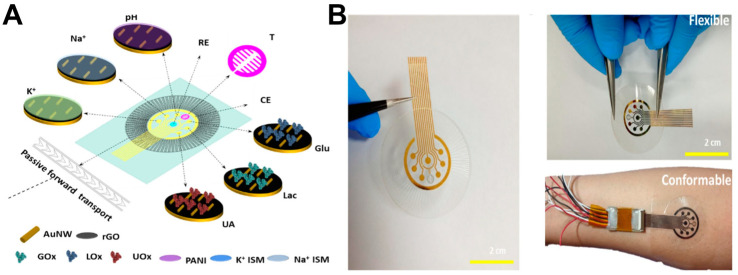
Flexible and wireless wearable biosensor patches for multiplexed monitoring of chronic wounds. (**A**) Schematic of the multiplexed electrochemical sensor array for metabolites and ions with the pH and temperature sensors. (**B**) Images of the fabricated wearable patch with flexibility and conformability with respect to the skin [70]. Copyright 2024 Elsevier.

**Figure 7:**
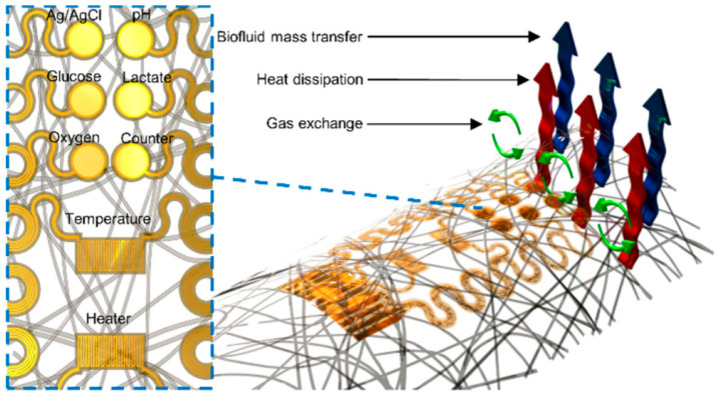
Schematic illustration of e-ECM for chronic wounds and cross-sectional view [71]. Copyright 2022 American Chemical Society.

Reza et al. [70] developed an integrated flexible multi-modal biosensing patch (CW-care patch) featuring microfluidic channels (Figure 6). This platform enables synchronous detection of seven key wound biomarkers: glucose (0–40 mM), lactate (0–4 mM), uric acid (0–150 µM), sodium ions (133–146 mM), potassium ions (3.2–5.7 mM), pH (4.0–9.0), and temperature (25–40 °C). It successfully addresses core challenges in chronic wound monitoring, including low efficiency in minimally invasive sampling, difficulty in synchronous multi-parameter detection, and compromised sensing accuracy due to the complex wound microenvironment. Experimental results demonstrated that the patch enhances wound exudate collection efficiency by 6.5-fold through its bioinspired barbed microneedle microfluidic structure. A pH-temperature compensation algorithm is employed to significantly improve detection accuracy. Utilizing this patch, successful continuous, wireless, and real-time monitoring of multiple biomarkers was achieved in diabetic rat wound models, enabling precise reflection of dynamic physio-logical changes throughout the infection-to-healing continuum. In a complementary approach, S. Brown et al. [71] developed an integrated flexible multi-modal biosensing patch (CW-care patch) featuring an adhesive-free, stretchable, and breathable multifunctional wound care platform (e-ECM) (Figure 7). This system enables synchronous monitoring of lactate (0–30 mM), glucose (0–8 mM), pH (5–10), dissolved oxygen (0–3.35 mL/L), and temperature, while integrating a heating element for wound thermoregulation. It effectively addresses critical limitations of conventional electronic devices, including rigidity, lack of breathability, adhesive-induced damage to newly formed tissue, and inability to monitor the dynamic physicochemical microenvironment of wounds in real time. Experimental validation demonstrated quantitative multi-analyte detection in both phosphate-buffered saline and authentic wound exudate. The microfilament-structured platform facilitates bidirectional exchange of wound exudate and gases while eliminating skin tearing risks through its adhesive-free design. Collectively, e-ECM provides an integrated monitoring solution for chronic wounds characterized by high breathability, mechanical compliance, and long-lasting conformal contact.

pH is another critical biochemical parameter regulating healing processes including collagen synthesis, inflammation, and angiogenesis [72]. Normal skin and acute wounds typically maintain an acidic microenvironment (pH 4.0–6.5), that promotes fibroblast proliferation, tissue regeneration, oxygen delivery, and a healthy microbiome [73,74]. This mild acidity also suppresses bacterial growth through neutrophil-mediated antimicrobial mechanisms [75]. In contrast, chronic wounds become alkaline (pH 7.0–10.0) due to ischemia-reperfusion injury, thereby leading to bacteria breeding. Severe infection can further raise the pH to values approaching 10.0 [76]. Electrochemical pH sensors detect wound pH through changes in potential, impedance, or Faradic current. Mariani et al. developed a textile-based smart bandage for real-time pH monitoring correlated with healing stages [77]. The system incorporated a dual-terminal pH sensor functionalized with semiconducting polymer and iridium oxide particles, along with an absorbent layer that continuously delivers wound exudate to the sensing surface. The electron conductivity of the poly(3,4-ethylenedioxythiophene): poly (styrene sulfonate) (PEDOT: PSS) charge-transport layer was reversibly modulated by spontaneous electrochemical reactions with analytes at the electrochemical transducer surface. The sensor exhibited a reversible response and linear response across the clinically relevant pH range (6–9), with a high sensitivity of (59 ± 4) μA pH^−1^.

**Figure 8:**
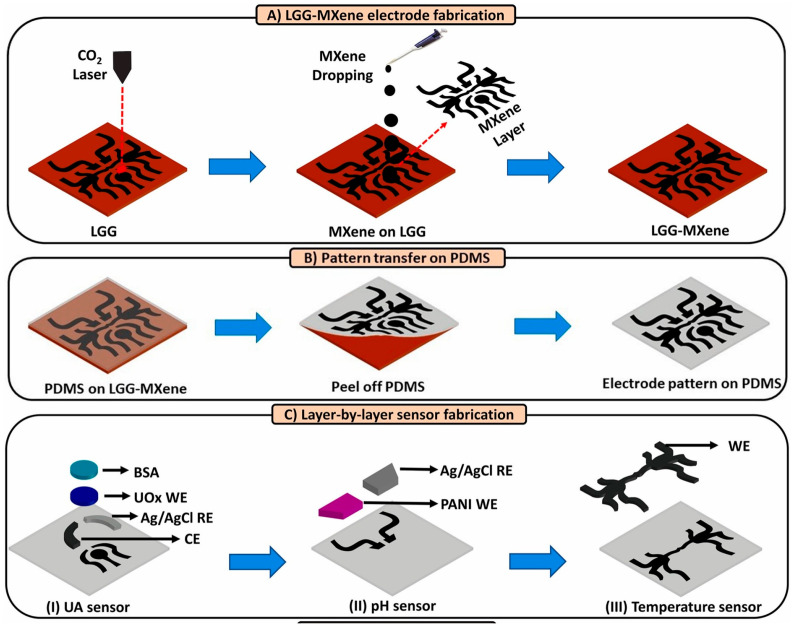
Stepwise fabrication scheme of the proposed stretchable and flexible smart bandage with an integrated multifunctional sensor. (**A**) LGG-MXene electrode fabrication. (**B**) Transferring the pattern onto PDMS using the peel-off process. (**C**) Layer-by-layer sensor fabrication (I) UA sensor, (II) pH sensor, and (III) temperature sensor [80]. Copyright 2020 Elsevier.

Uric acid (UA), which is specifically metabolized by microbial uricase, represents a distinctive biomarker for bacterial infection in wounds [78,79]. In wound exudate, UA exists primarily as urate, and the concentration is in a range from 220 μM to 750 μM. Unlike the common bacteria such as Staphylococcus aureus and Pseudomonas aeruginosa, which express uricase and actively catabolize UA, humans lack endogenous uricolytic enzymes. Consequently, a decline in UA levels below 200 μM serves as a metabolic signature indicative of bacterial colonization and infection. Given this, Sharifuzzaman et al. [80] designed a smart, stretchable, multifunctional sensing patch integrated within a wound bandage for detecting UA, pH, and temperature at the wound site (Figure 8). A highly conductive LGG-MXene hybrid scaffold was formed via covalent C-O-Ti crosslinking. This intelligent bandage provided valuable, accurate wound insights, guiding the design and regulation of the personalized treatment strategies.

Glucose monitoring provides critical insight for wound healing, particularly in diabetic chronic wounds where interstitial glucose levels fluctuate significantly (0–1.2 mM) [81]. Hyperglycemia suppresses the expression of hypoxia-inducible factor-1α (HIF-1α), a key transcription factor regulating cytokine activity and oxygen management during early wound healing [82,83]. This metabolic dysregulation impairs angiogenesis and promotes tissue necrosis. Furthermore, elevated glucose concentration in wounds also fuels bacterial proliferation [84]. Therefore, continuous glucose monitoring at the wound site has both diagnostic and therapeutic value.

ROS levels serve as sensitive indicators of infection and chronic inflammation, with elevated levels strongly correlating with impaired healing progression [85]. Oxygen concentration is another critical parameter in wound repair, as adequate tissue oxygenation is essential for cell proliferation, angiogenesis, and collagen deposition. Optimal oxygen levels facilitate macrophage recruitment, microbial defense, and growth factor delivery, whereas hypoxia severely impedes tissue repair [86]. Continuous oxygen monitoring guides the timely interventions, such as targeted oxygen therapy, to accelerate the wound healing.

Microbial-derived compounds provide direct evidence of pathogen colonization in wounds. Specific pathogens produce characteristic signatures, for example, Pseudomonas aeruginosa produces pyocyanin, while Staphylococcus aureus secretes virulence factors such as phospholipase A2 and α-hemolysin [87]. Monitoring these pathogen-specific metabolites enables early detection of wound infection.

For accurate wound assessment, biomarker selection should prioritize those demonstrating strong correlation with specific pathologies (Table 1). Repeated measurements and multi-modal monitoring are crucial, as reliance on any single parameter provides misleading diagnostic information. Comprehensive evaluation of multiple interrelated biomarkers offers the most reliable foundation for clinical decision-making in wound management.

### 4.2. Early Detection of Wound Infection

Early detection of wound infection is critical for preventing severe complications such as sepsis and tissue necrosis, and for improving therapeutic outcomes [88]. Biomarkers—which are quantifiable indicators of biological processes—are versatile tools in healthcare. Their integration into wound detection, diagnosis, and continuous monitoring significantly enhances assessment precision. Wearable biosensors represent powerful platforms that facilitate real-time, non-invasive wound monitoring by targeting specific biomarkers, including bacterial metabolites, enzyme activity, and dynamic inflammatory mediators.

**Figure 9:**
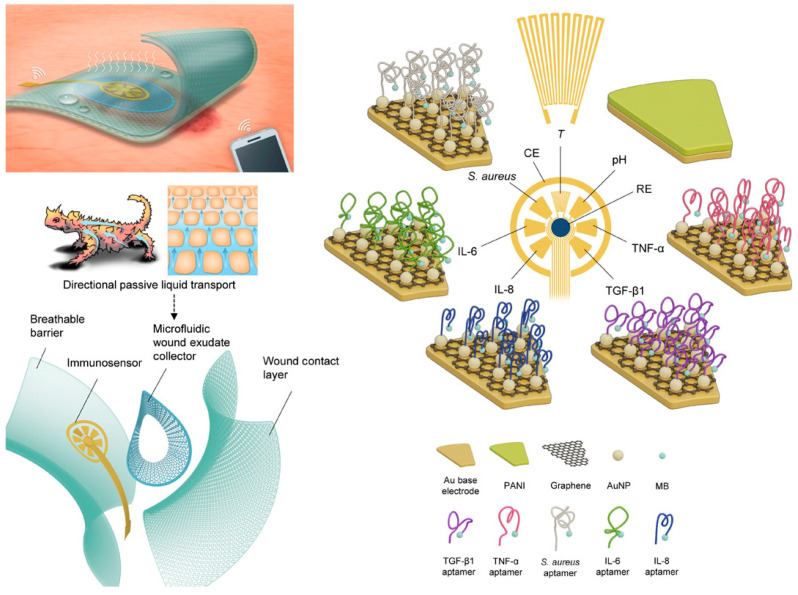
Schematic of a multiplexed immunosensing system for chronic wound monitoring. Schematic of the immunosensor for detection of TNF-α, IL-6, IL-8, TGF-β1, S. aureus, pH, and temperature. PANI, polyaniline; MB, methylene blue; RE, reference electrode; CE, counter electrode [90]. Copyright 2021 American Association for the Advancement of Science.

Inflammatory biomarkers, such as interleukins (IL-6, IL-8), C-reactive protein (CRP), and TNF-α, serve as reliable indicators of immune activation within the wound microenvironment [89]. Their detection typically relies on highly sensitive and specific biosensing strategies, such as immunoassays or electrochemical sensing. Recent advances in wearable patches integrated with microfluidics enable continuous exudate collection and real-time cytokine monitoring. Persistent elevation of these markers indicates chronic inflammation or underlying infection, highlighting the need for clinical intervention. Leveraging these advanced wearable biosensing technologies facilitates early infection warning, while significantly reducing the risk of complication. Gao et al. [90] developed an MXene-functionalized porous laser-induced graphene (LIG) scaffold-based smart bandage integrating uric acid (UA), pH, and temperature sensors (Figure 9). By constructing a hybrid LGG-MXene scaffold through C-O-Ti covalent crosslinking between 2D MXene nanosheets and 3D LGG frameworks, they significantly enhanced electrical conductivity and electrochemical performance, subsequently transferring the scaffold to a PDMS substrate to yield a stretchable, flexible multifunctional sensor-integrated bandage. The UA sensor exhibited 422.5 μA mM^−1^ cm^−2^ sensitivity within 50–1200 μM (LOD: 50 μM); the pH sensor demonstrated linear Nernstian behavior at ~57.03 mV pH^−1^ (pH 4.0–9.0); while the temperature sensor achieved 0.09% °C^−1^ sensitivity (R^2^ = 0.999) across 25–50 °C. This platform enables real-time, accurate monitoring of key wound biomarkers and holds promise to revolutionize wound care management with profound therapeutic impact.

**Figure 10:**
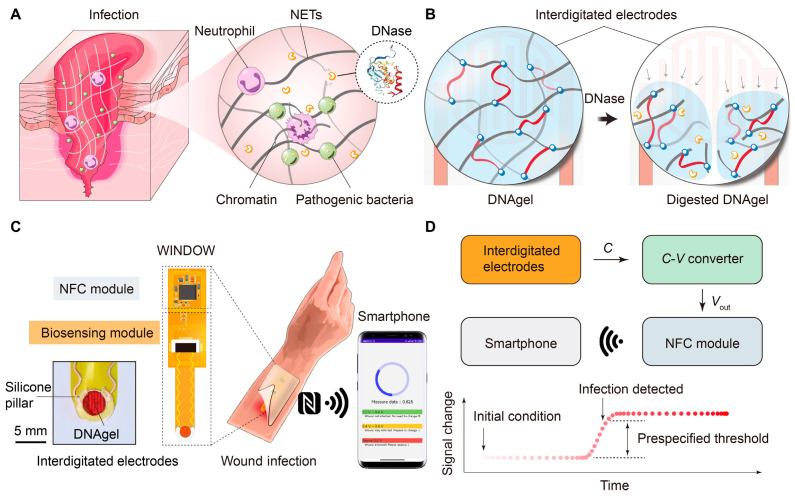
WINDOW concept. (**A**) DNase is a virulence factor in wound infections. Pathogenic bacteria secrete DNase to evade neutrophil extracellular traps (NETs), which are integral to the host’s immune response. (**B**) Schematic of the infection-sensing mechanism. DNAgel is degraded upon exposure to DNase, resulting in a change in the capacitance of the sensor. (**C**) Schematic of the wireless wound infection sensor. WINDOW integrates the bioresponsive DNAgel, a half-wave-rectified LC biosensing module, and an NFC module to enable smartphone readout of the wound status. Inset image: Sensor-integrated DNAgel stained with rhodamine B. (**D**) System block diagram showing signal transduction from the DNAgel-based biosensor to the NFC module and to a smartphone for wireless readout and display [91]. Copyright 2021 American Association for the Advancement of Science.

Additionally, detecting specific microbes is crucial for evaluating wound bioburden and infection status. For example, Xiong et al. [91] developed a wireless, battery-free wound infection sensor (WINDOW) based on DNA hydrogel (Figure 10). This sensor enables early detection of the pathogenic bacterium Staphylococcus aureus via smartphone near-field communication (NFC) technology. The detection mechanism leverages the DNA hydrogel’s specific response to deoxyribonuclease (DNase) secreted by pathogens. Following enzymatic degradation of the hydrogel by DNase, alterations in its dielectric properties induce measurable changes in capacitance, which are subsequently converted into a wireless signal output (Figure 10A). When exposed to extracellular DNase, the DNAgel is degraded via nonspecific cleavage of DNA strands, resulting in dissolution of the hydrogel. This changes the dielectric permittivity of the region above an interdigitated electrode, thereby modulating its capacitance (Figure 10B). By connecting the electrode to an embedded system, this electronic signal can be read out in a wireless and battery-free manner using near-field communication (NFC), a connectivity technology found on most modern smartphones for short-range communication and wireless power transfer (Figure 10C). WINDOW has a thin and flexible form factor that enables it to be conformally embedded into wound dressings to wirelessly track virulence factor activity on demand (Figure 10D).

Intelligent bandages incorporating integrated microelectronic sensors provide a transformative approach to chronic wound management by continuous, multi-parameter monitoring of key physiological indicators, such as temperature, pH, metabolites, tissue oxygen, inflammation markers, and microbial presence [92]. They enhance clinical responsiveness by providing real-time data on wound status, enabling accurate tracking of healing progression, reducing dependence on frequent in-person clinical assessments, and facilitating early detection of complications such as infection and impaired perfusion. As a result, timely interventions can be implemented to mitigate severe infection risks and improve healing outcomes [93]. Furthermore, the continuous acquisition of multi-parameter data also supports the development of personalized, dynamically adaptive treatment strategies.

Intelligent bandage systems allow patients to maintain daily activities with minimal disruption while reducing the frequency of clinic visits. Through proactive and continuous monitoring, these systems facilitate early detection of abnormalities, allowing for timely interventions that lower complication rates, accelerate the healing process, and decrease overall treatment costs. Although the initial expense may be higher, long-term economic benefits are anticipated due to reduction in hospitalizations and complication treatments. Furthermore, seamless integration with telehealth platforms permits remote wound assessment and medical guidance by clinicians. The accumulated data deepens the understanding of wound healing mechanisms and supports the development of novel therapeutic strategies. In summary, by facilitating multiple monitoring of biomarkers and responsive intervention, intelligent bandage technology significantly enhances the precision and prognostics of chronic wound management.

### 4.3. Smart Sensing-Actuating Platforms for Intelligent Wound Management

**Figure 11:**
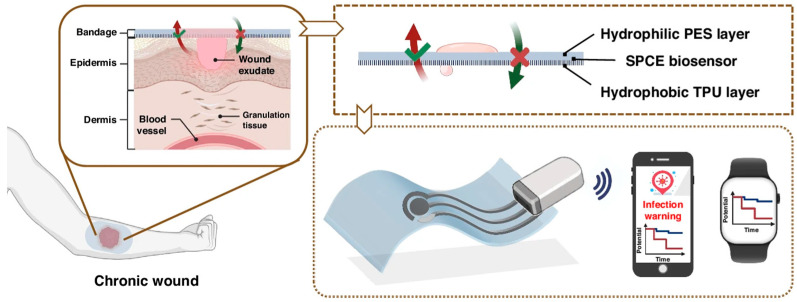
Schematic illustration of the design and fabrication of the integrated smart bandage (“√” indicates permission/normality/normal operation, while “×” indicates prohibition/abnormality/failure of the function) [94]. Copyright 2024 Elsevier.

Therapeutic feedback systems integrate wearable biosensing platforms with controlled drug delivery mechanisms to dynamically detect physiological changes during wound healing, enabling precise therapeutic interventions tailored to the wound’s dynamic needs. The emergence of intelligent wound dressings is transforming the traditional clinical paradigms by facilitating multi-data-driven decision-making and improving management efficacy. In 2025, Wang et al. [94] introduced an intelligent wound management bandage incorporating a biocompatible liquid-diode membrane and an ultrasensitive pH biosensor based on 3D mesh polyaniline (M-PANI) (Figure 11). This integrated bandage enabled unidirectional exudate drainage/removal and dynamically monitored wound pH, providing real-time insights into the healing process. The M-PANI sensor showed high sensitivity (61.5 mV/pH) across a broad pH range (4.0–10.0), covering physiologically relevant ranges. This system also demonstrated high stability, with less than 4.8% signal decline during 48 h of continuous operation and only 3.1% relative standard deviation (RSD) over 28 days of storage. The system shows considerable potential for home-based wound care. Despite such promising advances, systems reliant on a single biomarker remain limited in diagnostic accuracy and are susceptible to misinterpretation. Therefore, further efforts should focus on the integration of multi-analyte monitoring platforms to enhance diagnostic accuracy and therapeutic reliability. In this regard, Wang et al. [95] developed an integrated Janus bioelectronic wound dressing composed of an electrosprayed Janus nanofiber membrane, featuring AgNP-impregnated thermoplastic polyurethane, and a hydrophilic gauze layer. This dressing could provide antibacterial protection and active exudate transport. Coupled with laser-engraved graphene electrochemical sensors for detecting glucose, UA, and pH, this system enabled real-time assessment of inflammatory status. The asymmetric wettability structure facilitated efficient pumping of wound exudate to external sensors, ensuring continuous monitoring. The multifunctional bandage combined exudate management, antibacterial therapy, and continuous biomarker monitoring, demonstrating high sensing performance with a detection limit of 0.15 mM for glucose, 6.85 μM for UA, along with a pH sensitivity of 60.76 mV/decade within the pH range of 4–8. In a murine full-thickness wound model, the dressing promoted wound healing, achieving 90.35% wound closure by day 14, and successfully dynamically tracked the three biomarkers over 3 days.

**Figure 12:**
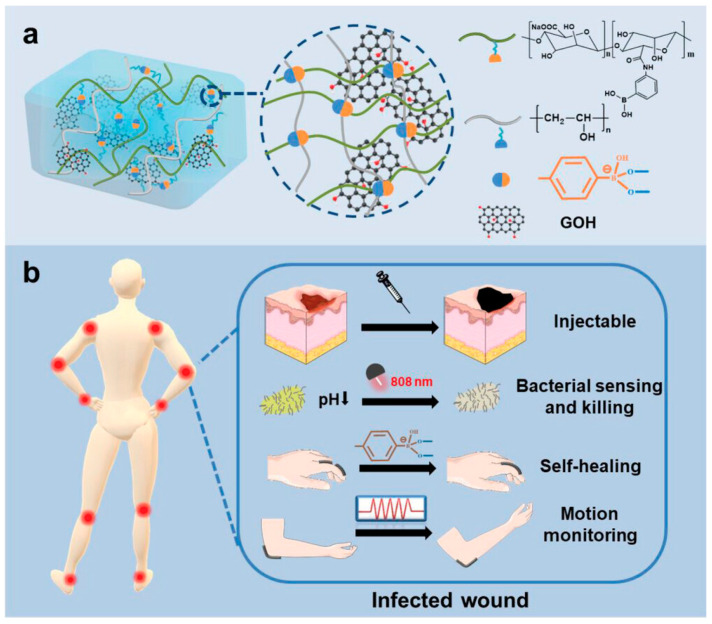
Schematic representation of the crosslinked network (**a**), application and multi-functions of Alg-PBA/PVA/GOH hydrogels (**b**) (↓ indicates a decrease in local pH due to bacterial infection) [99]. Copyright 2024 Wiley.

Clearly, the adoption of NFC for wireless power transfer and data communication enables battery-free operation, significantly minimizing PCB size and enhancing wearability and patient compliance of wound dressings [96]. However, traditional sensors and conductive hydrogel-based sensors remain susceptible to physical or mechanical damage within the complex wound environments, particularly under prolonged use and patient movement, which will compromise their stability and operational reliability [97,98]. A promising strategy to overcome this challenge involves the utilization of self-healing materials that autonomously recover their structural integrity and electrical functionality without external stimuli. In view of this, Shan et al. [99] reported a multifunctional, stretchable injectable hydrogel with self-healing properties, motion monitoring, in situ bacterial sensing, and non-antibiotic bactericidal activity for infected joint wounds (Figure 12). The hydrogel, fabricated through supramolecular interactions of amino phenyl boronic-acid-grafted sodium alginate (PBA-Alg), polyvinyl alcohol (PVA), and hydroxylated graphene, exhibited rapid self-healing (<60 s) and integrated capabilities for in situ bacterial biosensing, non-antibiotic bactericidal action, and joint motion tracking. The system detected bacteria within 15 min via electrical signal perturbations and achieved >99% antibacterial efficiency through photothermal therapy, thus avoiding antibiotic resistance. In vivo studies demonstrated accelerated wound healing. Additionally, its strain-responsive mechanoelectrical properties enabled real-time joint motion monitoring and biomechanical feedback to prevent re-injury.

In summary, intelligent systems that combine wearable electrochemical sensing with actively therapeutic dressings represent a cutting edge in chronic wound therapy. In Table 2, we have compared the application concepts of wearable electrochemical sensors. Integrating real-time diagnostics, early detection, and closed-loop therapeutic intervention significantly enhances the precision and proactivity of wound management. These advanced platforms offer a transformative strategy for addressing the complex clinical challenges associated with refractory wounds and hold significant potential to improve healing outcomes.

## 5. Challenges and Limitations

### 5.1. Technical Challenges

Despite considerable promise, the practical implementation of wearable electrochemical sensors for wound management faces several critical technical challenges. The key limitations include measurement accuracy under dynamic physiological conditions, detection sensitivity in complex biofluids, and a high stable power source for continuous monitoring, as well as long-term stability under real-world conditions.

Analytical accuracy, which is fundamental to device reliability, remains a paramount concern in wearable electrochemical sensing [100]. Although high accuracy is theoretically achievable for biomarkers such as pH, temperature, and specific cytokines, practical accuracy is often undermined by signal drift and interference from co-existing species in complex biofluids. To improve specificity and minimize non-specific binding and cross-reactivity, advanced functional interfaces, including molecularly imprinted polymers and antibody-modified electrodes, have been incorporated into sensor designs [101]. However, these materials often require complex, costly fabrication processes, and their generalizability across different patient/wound types has not yet to be thoroughly validated. Moreover, the highly dynamic nature of the wound microenvironment persistently challenges the physicochemical stability of substrate materials and efficiency of functional layers, posing a persistent barrier to broad clinical application.

Sensitivity determines the lowest detectable concentration of target biomarkers, which is critical for the early identification of infection and the tracking of subtle physiological changes during healing [102]. Achieving high sensitivity typically requires sophisticated sensor architectures (e.g., micro-or nano-electrodes) or the use of signal amplification nanomaterials (e.g., graphene, metal nanoparticles) [103]. However, these strategies introduce practical challenges: complex nanofabrication processes increase production costs and manufacturing complexity; the stability of such nanomaterials in harsh, moist wound environment is often limited, leading to material degradation, aggregation, or gradual loss of function. Furthermore, high-sensitivity designs are frequently accompanied by increased susceptibility to noise and non-specific interactions, particularly at low analyte concentrations. Compensating for these limitations requires sophisticated signal processing, which further complicates their use in cost-effective, wearable applications.

Durability is a critical limiting factor for the sustained, reliable operation needed for chronic wound monitoring [104]. A primary restriction is energy supply. Most current systems depend on external batteries, which impede uninterrupted and unobtrusive operation. And replacing batteries requires device removal, interrupting monitoring continuity and causing secondary harm to the wounds. While energy harvesters offer a promising alternative, they face substantial hurdles related to power insufficiency and unstable power output [105]. Furthermore, sensors operating in direct contact with the wound suffer from performance degradation due to the mechanical stress (flexing, pressure), biological fouling (protein/ECM deposition), and chemical corrosion [106]. For instance, the functional lifespan of many electrochemical sensors is limited to several days or weeks due to electrode passivation, denaturation of biological recognition elements, and accumulated biofouling. Considering that even acute wounds require 2–4 weeks to heal and chronic wounds may persist for months, current sensor durability falls significantly short of clinical needs.

Current efforts have been made to overcome the above-mentioned challenges through several key aspects: developing novel materials with intrinsic self-healing and antifouling properties; implementing robust encapsulation/barrier layers; and exploring long-term stable self-powered mechanisms (e.g., robust BFCs, hybrid energy harvesting systems) [107]. For electrochemical sensors, innovative strategies are being pursued to enhance operational longevity and stability, including novel enzyme immobilization techniques, degradation-resistant nanocomposites, and ultra-low-power sensing circuitry [108].

While considerable advances have been made, wearable electrochemical sensors remain largely in the laboratory-to-clinic transition phase. Realizing the full potential of intelligent wound management will require a paradigm-shifting, multidisciplinary integration of materials science, microelectronics, and biomedicine. Such interdisciplinary integration is essential to achieve next-generation systems that are biodegradable, long-term operational, energy self-sustained, clinically accurate, and ultimately practical for effective wound care.

### 5.2. Clinical Translation

Beyond technical obstacles, the widespread clinical adoption of wearable biosensors for wound management confronts significant non-technical barriers, including regulatory approval, patient adherence, cost-effectiveness, and data privacy. Regulatory clearance represents the foremost translational barrier. Regulatory agencies such as the U.S. Food and Drug Administration (FDA) and the European Medicines Agency (EMA) impose stringent requirements on safety and efficacy for Class II/III medical devices [109]. A major challenge is generating the substantial validation data required to demonstrate device biocompatibility, long-term stability under both simulated and real-world conditions, performance consistency throughout operational and shelf life, biocompatibility, and ultimately, clinical utility. These demands typically necessitate large-scale clinical trials, a process that is protracted and resource-intensive, significantly increasing the development burden. Patient adherence is critical for the efficacy of long-term wound monitoring using wearable biosensors [110]. To encourage consistent use over an extended period, the sensors must be designed to be comfortable, inconspicuous, and user-friendly. Suboptimal design that causes skin irritation, physical discomfort or frequent maintenance significantly diminishes compliance, particularly among elderly or chronically ill populations. It is essential that systems operate with minimal maintenance and offer user-friendly interfaces. Addressing these challenges requires coordinated collaboration among researchers, clinicians, and regulatory agencies to accelerate regulatory approvals, reduce costs, and promote the development of truly patient-centered systems.

Cost and accessibility present a substantial barrier to the widespread implementation of wearable wound biosensors. The use of advanced sensing technology and specialized functional materials often leads to high manufacturing costs, thereby resulting in prohibitively expensive end products. This limits accessibility, particularly in low-resource healthcare settings or patients with limited insurance coverage. Failure to control development and production costs effectively will impede broad adoption.

Animal models play an indispensable role in the development and validation of wearable electrochemical sensors. Mouse excision wound models, which emulate key aspects of human inflammatory and healing responses, are widely used to dynamically track key biomarkers (e.g., pH, specific cytokines, ROS) to provide in vivo validation of sensor functionality. Diabetic rat and porcine wound models are particularly valuable for simulating the complex pathology of diabetic ulcers and highly susceptible infected chronic wounds, yielding vital data on sensor reliability under clinically analogous conditions. Furthermore, in vitro biomimetic models enable controlled, high-throughput assessment of sensor sensitivity and specificity during initial development. Due to ethical considerations and regulatory restrictions on early human tests, these predictive preclinical models constitute an indispensable phase in the pathway towards clinical translation.

### 5.3. Power Management

Sustainable power management remains a central challenge in the development of wearable biosensors [111]. Although advances in ultra-low-power electronics and wireless energy harvesting have reduced the need for frequent recharging and improved user convenience, developing reliable and compact energy systems, especially for self-powered or battery-free sensing, remains technically difficult. Current systems harvest energy from ambient sources, such as body heat, motion, or biochemical energy from biofluids, but their practical application is constrained by limited power density, low conversion efficiency, and insufficient long-term stability under conditions of dynamic movement and environmental fluctuations [112].

Energy harvested from motion or biochemical sources is typically low and intermittent, which is insufficient to support the continuous operation of high-energy-consuming sensors that require real-time signal processing or frequent wireless data transmission. Similarly, battery-free architectures eliminate the need for battery replacement and reduce electronic waste, but they are challenged by the inherent intermittency of ambient energy sources and the limits on energy storage capacity. Current miniaturized energy storage units struggle to deliver sustained power without substantially increasing the device’s size and weight, consequently, compromising wearability and functionality.

Effective intelligent power management represents a critical and unresolved challenge in the development of autonomous wearable biosensors [113]. Sensors must autonomously modulate their operational parameters, such as sensing frequency, computational load, and data transmission rate, according to the real-time energy availability and the clinical relevance of the data, to prolong operational life under conditions of constrained energy availability. Overcoming these energy constraints necessitates synergistic advances in ultra-low-power integrated circuit design, the development of high-efficiency energy harvesting and storage materials, and the power-gating strategies aligned with genuine clinical scenarios and user-centered design principles. Such integrated efforts are essential to realize the next generation of self-sustaining and clinically viable biosensors.

## 6. Advancing Technical Solutions: Addressing Key Barriers in Wearable Wound Sensing Technology

### 6.1. Multimodal Sensing and Signal Enhancement

**Figure 13:**
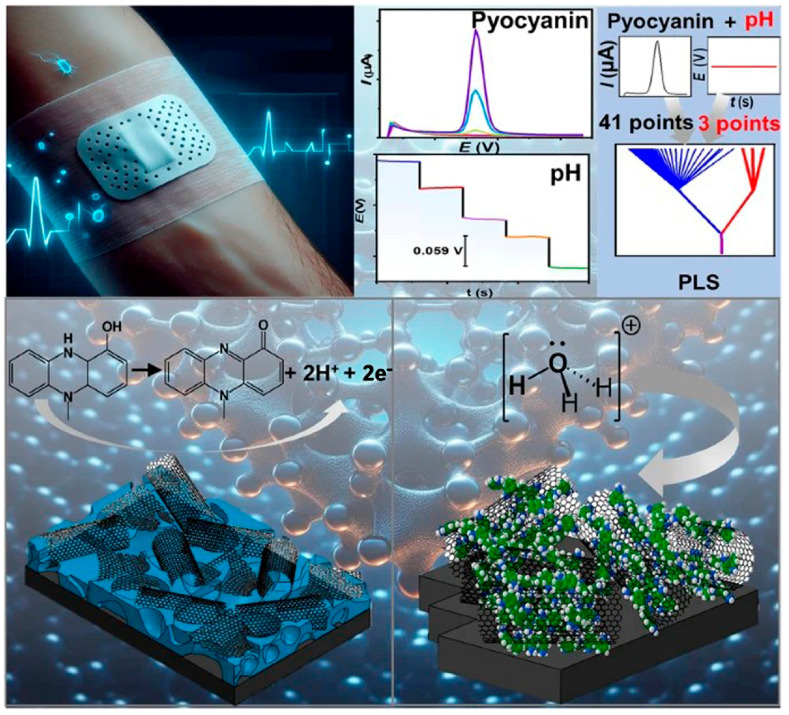
The conceptual presentation of the bandage-based sensing array for determining pyocyanin and pH in a wound with a pH-correction system for infection monitoring (comparison of predicted pyocyanin concentrations using the traditional regression method (blue) and our proposed method (pink) for pyocyanin concentration analysis at different pH values) [113]. Copyright 2024 Springer Nature.

Constructing a multimodal sensing array represents a core strategy for enhancing sensor accuracy in dynamic wound environments. Such systems enable the simultaneous acquisition of multiple parameters critical to wound status, including exudate temperature, pH, ionic strength, and inflammatory marker concentrations. By correlating target analyte responses with these environmental parameters in real time, it becomes feasible to model and compensate for key microenvironmental fluctuation-induced interferences—such as enzyme activity variations and deviations in receptor-ligand binding constant deviations. The implementation of correction algorithms based on partial least squares regression (PLS) allows for the analysis of unpredictable chemical shifts within biological fluids, thereby significantly reducing pH fluctuation-induced errors in monitoring wound infection status. Furthermore, the design of microfluidic systems is critical for controlling sample evaporation and preventing cross-contamination. An optimized microchannel configuration enables the continuous extraction of fresh wound exudate while isolating degraded or contaminated fluids, thereby ensuring that the sensing interface consistently interacts with representative samples. This functionality substantially enhances data consistency and reliability. For instance, Kaewpradub et al. developed a screen-printable conductive nanocomposite ink for fabricating the pyocyanin sensor and utilized a polyaniline/carbon nanocomposite as a pH-sensitive film for the pH sensor (Figure 13) [113]. The combination of both nanomaterial-based sensors yielded a porous pyocyanin-sensitive transducer and a Nernstian-sensitive pH sensor. To mitigate the influence of pH fluctuations, the authors developed a mathematical pH-correction model using partial least squares regression (PLS), effectively addressing this challenge. By overcoming the critical limitation associated with pH sensitivity, this design bridges a gap in monitoring technology, aiming to improve diagnostic accuracy and support personalized patient care pathways.

A critical challenge lies in enhancing the detection sensitivity of key wound biomarkers—such as cytokines and growth factors—present in wound exudate. To address this, multiple signal amplification strategies have been employed, including nanomaterial modification, enzymatic catalytic amplification, and interface engineering of biomolecular probes [114]. Numerous studies have demonstrated effective electrochemical biosensor approaches for wound monitoring, utilizing structural optimization of recognition elements, enzyme cascade reaction systems, and high-loading-capacity nanomaterials (e.g., gold nanoparticles and 2D transition metal sulfides). These methods collectively enhance sensitivity by several orders of magnitude through increased density of biological recognition elements and accelerated electron transfer. Gong et al. developed a novel electrochemical biosensor for detecting Matrix Metalloproteinase-2 (MMP-2), a critical biomarker in chronic wound diagnostics [115]. This biosensor integrated a programmable RNA detection system with the synergistic amplification capabilities of T7 RNA polymerase and CRISPR/Cas12a. In the analysis of chronic wound fluid, the platform achieved an ultralow detection limit of 0.496 fM MMP-2, demonstrating sufficient sensitivity to quantify pathologically relevant enzyme concentrations. This capability enables the early identification of dysregulated protease activity—a key indicator of stalled healing in pressure ulcers and diabetic wounds.

**Figure 14:**
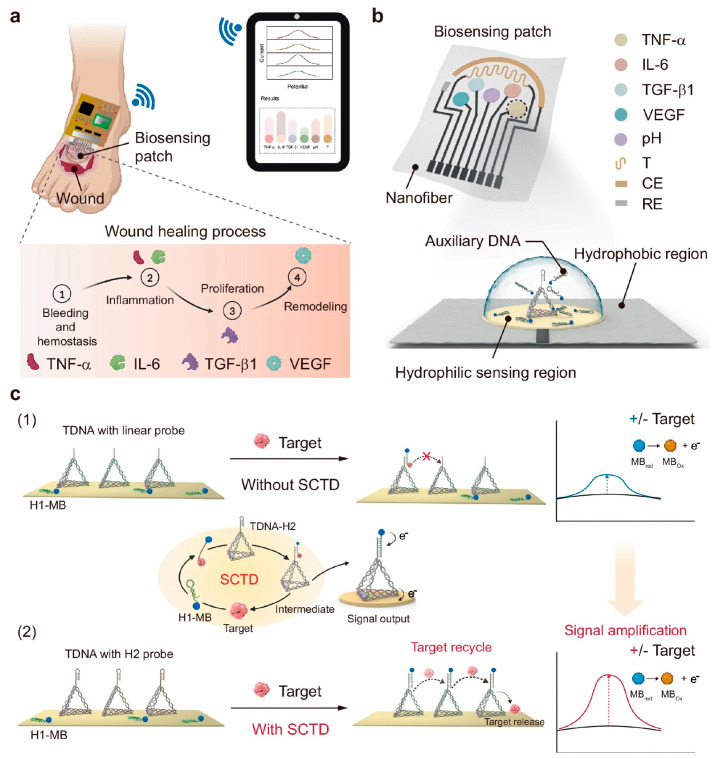
Design of the integrated wearable biosensing system (**a**): schematic of a wearable, breathable, and wireless FPCB on a chronic diabetic foot wound; wound healing mechanism and different wound healing-related proteins released in different stages of wound healing. Created in BioRender. (**b**): schematic illustration of the nanofiber-based multiplexed biosensing electronics, which comprises biophysical and biochemical sensors for pH, temperature, and wound healing-related proteins detection. Bottom: DNA solution confined in the hydrophilic region of the nanofiber based on patterned wettability. T temperature, CE counter electrode, RE reference electrode. (**c**): schematic comparison of DNA circuit reactions and sensing signals triggered by target with (2) and without (1) SCTD, along with illustration of SCTD working principle: In hydrophilic-confined regions, target proteins in wound exudate trigger DNA cascade amplification around electrode-anchored TDNA, with pre-coated auxiliary DNA (H1) dissolution enabling the amplification process [116]. Copyright 2025 Springer Nature.

For electrochemical biosensors deployed in wound environments, biofouling at the electrode surface (e.g., protein adsorption, cell adhesion) poses a major challenge. This issue not only significantly compromises detection sensitivity and accuracy, but also drastically shortens device lifespan due to significant signal drift and interface degradation. To address this challenge, a variety of antifouling interfacial engineering techniques have been developed, enabling sensors to maintain operational stability in the complex wound microenvironment saturated with exudates, while effectively countering adverse effects such as hydrolysis, oxidation, and enzymatic degradation. Zhao et al. [116] developed a soft bioelectronic platform embedded with a Self-Confined Tetrahedral DNA Circuit (SCTD) for wound monitoring. The integrated wearable biosensing system consists of a multimodal biosensing array and a miniaturized flexible printed circuit board (FPCB) for signal processing and wireless communication with the user interfaces. The concentration of growth factors, particularly vascular endothelial growth factor (VEGF), essential for angiogenesis and tissue repair, increases during the wound healing process (Figure 14a). To capture these dynamic changes, a multimodal biosensor array was integrated into the wireless system for simultaneous detection of wound healing-related proteins and biophysical parameters, reflecting the state and stage of wound healing, without causing physical damage or impeding the healing process (Figure 14b). Proteins in wound exudate trigger a spatially confined DNA self-circulation amplification within hydrophilic zones, reducing detection limits by one order of magnitude (Figure 14c). The tetrahedral DNA scaffold provides exceptional mechanical stability (<3% variation after 1000 bending cycles), extended operational stability (<8% signal attenuation over 4 weeks), and reduced biofouling (>50% decrease in BSA adhesion). Integrated with wireless readout capabilities, the platform simultaneously monitors multiple wound-healing biomarkers (TNF-α, IL-6, TGF-β1, and VEGF) alongside biophysical parameters. Furthermore, the inherent anti-degradation and antifouling properties of the TDNA prevent degradation of biosensing elements and biofouling in the complex wound microenvironment, exhibiting long-term stability for up to four weeks. By integrating biochemical and biophysical sensing arrays, a wireless FPCB, and a smartphone-based user interface, this breathable sensing system allows for in situ, real-time, sensitive, and stable monitoring of multiple physiological indicators at the wounds of diabetic mice, without impeding the healing. This biosensing platform can facilitate a comprehensive assessment and provide treatment guidance for chronic wounds.

### 6.2. Self-Powered Sensing and Active Therapeutics

**Figure 15:**
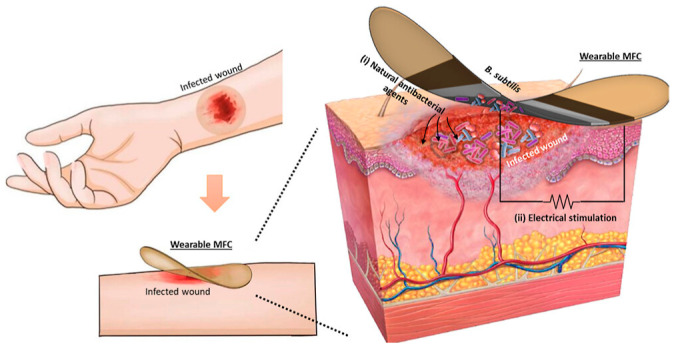
Integrated MFC systems as a revolutionary solution of smart wound dressings (the underlined part indicates wearable microbial fuel cell framework) [119]. Copyright 2024 American Chemical Society.

Despite their advantages of compact structure, ease of integration, and low cost, traditional electrochemical biosensors are constrained by limited sensitivity and a strong dependence on external power sources, which consequently restricts their capability for continuous and accurate monitoring of biomarkers in complex physiological microenvironments [117]. Therefore, self-powered, autonomous sensing systems represent a pivotal research frontier in wearable electrochemical biosensing. Self-powered technologies that extract energy from the human body or the ambient environment are particularly promising due to their sustainability, biocompatibility, and potential for seamless integration [118]. Various energy technologies have been incorporated into wearable electrochemical sensors, including wearable batteries, supercapacitors, solar cells, biofuel cells (BFCs), thermoelectric generators, and piezoelectric/triboelectric nanogenerators. As a representative study, M. Rezaie et al. [119] demonstrated a novel dual-functional active dressing based on an advanced two-dimensional microbial fuel cell (MFC) architecture, which concurrently enabled autonomous antibacterial agent generation and electrical stimulation (Figure 15). The dressing incorporated dormant Bacillus subtilis spores as efficient dormant biocatalysts. Upon hydration by nutrient-rich wound exudate, the spores germinated. The activated cells then generated extracellular electricity through metabolic processes and secreted antibacterial agents to outcompete pathogens for resources. This combined therapeutic strategy demonstrated robust antibacterial efficiency against Pseudomonas aeruginosa, Escherichia coli, and Staphylococcus aureus, effectively eliminated the preformed biofilms and inhibited new formation. This approach represents a novel paradigm in the development of MFC-based wearable wound dressings, significantly enhancing the treatment of infected wounds.

### 6.3. Intelligent Telemedicine for Dynamic Wound Management

The large-scale implementation of wearable biosensors within telemedicine and personalized care frameworks holds immense potential for revolutionizing the management of wound healing and infection monitoring. Integrating this technology with telehealth platforms enables continuous wound status monitoring and data sharing, making specialist-level oversight accessible to patients in their homes. This capability is particularly transformative for managing chronic wounds like DFUs and pressure ulcers, significantly reducing hospital visits, lowering healthcare costs, and improving both quality of life and self-management adherence.

Wearable biosensors add a novel dimension to wound care by facilitating the remote, real-time tracking of critical physiological and biochemical parameters (e.g., pH, temperature, cytokine patterns, bacterial activity), thereby enhancing management efficiency for chronic, vulnerable wounds. These devices transmit data wirelessly to cloud-based platforms, allowing clinicians to remotely assess wound status, formulate personalized treatment plans, and dynamically adjust protocols, including adjustments to dressings, topical agents, and antibiotic regimens [120]. This integrated approach diminishes the need for frequent in-person consultations, thus decreasing healthcare costs and improving patients’ quality of life, especially in resource-limited settings.

For personalized wound care, wearable sensors generate temporally dense data of the wound microenvironment. When processed by ML models that are trained on individual healing trajectories, this continuous data creates a powerful foundation for dynamically optimizing treatment protocols through evidence-based and data-driven interventions. Further integration with AI enhances remote systems with predictive analytic capabilities, enabling early detection of infection risk or healing stagnation and triggering automated clinical alerts. This facilitates prompt and precisely targeted interventions [121].

## 7. Conclusions and Prospects

Wearable electrochemical biosensors represent a paradigm shift in chronic wound management through real-time, multi-parameter, high-precision monitoring of key biochemical and biophysical markers within the wound microenvironment, such as pH, temperature, dissolved oxygen, inflammatory cytokines, microbial metabolites, and ROS. This review comprehensively summarizes recent advancements in core technologies, including efforts toward energy autonomy through self-powered systems and enhanced energy harvesting and storage, the development of integrated closed-loop therapy systems combining intelligent drug release with electrical stimulation and multi-modal sensor fusion.

Despite their considerable advantages, the widespread clinical adoption of wearable electrochemical biosensors remains constrained by several critical challenges that demand innovative solutions. For example, substantial clinical translation hurdles persist, including critical concerns regarding data security, compliance with medical device regulatory standards, economic viability, and fostering acceptance among clinicians and patient groups.

Future research must systematically construct a dual-focus objective framework targeting both technological advancement and clinical translation. At the foundational hardware and system level, efforts should prioritize the development of next-generation biocompatible and biodegradable flexible sensing materials with enhanced environmental stability and integration potential, alongside designing sophisticated multi-source energy harvesting systems coupled with ultra-low-power electronics and intelligent energy management modules. For the optimization of core functionality, research should aim to enhance detection specificity and suppress interference through optimized biointerface engineering and novel multi-modal sensing designs that decouple overlapping signals, complemented by miniaturization technologies to facilitate seamless device integration. For clinical application expansion, the focus should be placed on building and validating robust ML-based dynamic prediction models capable of accurately predicting healing progression and complication risks, and demonstrating tangible improvements in patient outcomes, workflow efficiency, and cost-effectiveness across diverse wound types through rigorous in-human verification studies. Ultimately, establishing a comprehensive translation pathway necessitates clear regulatory approval pathways, scalable manufacturing processes, cost-reduction measures, and supportive reimbursement models.

Achieving the vision of personalized, intelligent, and remotely accessible management of chronic wounds requires deepened interdisciplinary collaboration across engineering, material science, computer science, clinical medicine, and health policy. Through such synergistic innovation driven by genuine clinical need, and validated via user-centered and real-world deployment, this technology holds immense promise for evolving beyond laboratory prototypes into widely adopted clinical tools. Such transformation promises not only to improve clinical outcomes for the global population affected by chronic wounds but also to significantly enhance healthcare efficiency and quality of life for these patients.

## Figures and Tables

**Table 1:** Clinical thresholds of key biomarkers for wounds and their correlation with pathological manifestations (↑ and ↓ indicate an increase or decrease).

Biological Markers	Detection Ranges	Reference Values for Health Status	Abnormal Threshold/Clinical Significance	Multi-Parameter Interlocking Value
Temperature	25–40 °C (Sensor range)	31.1–36.5 °C (Recovery of normal wound)	Below normal: ↓ 2.2 °C → Blood circulation disorder/Enzyme activity reduction/Lymphocyte decrease Above normal: ↑ 2.2 °C → Infection/Severe inflammation	Linked pH compensation for detecting errors
pH	4.0–9.0 (CW-care) 5–10 (e-ECM)	4.0–6.5 (Healthy skin/Acute wounds)	>7.0: Markers of chronic wound alkalization >10.0: Severe infection (such as alkaline-producing bacteria/MRSA biofilm) Pathological mechanism: Ischemia-reperfusion injury → Bacterial proliferation	Regulation of collagen synthesis/angiogenesis/pathogen inhibition
Uric acid (UA)	0–150 μM (CW-care)	220–750 μM (No infection status)	<200 μM: Indicator of bacterial infection	Different from the specific infection indicators of endogenous metabolism
Glucose	0–40 mM (CW-care) 0–8 mM (e-ECM)	Diabetic wound fluctuations: 0–1.2 mM	Hyperglycemia: Inhibits HIF-1α → Promotes angiogenesis; Facilitates tissue necrosis; Bacterial proliferation	The core metabolic indicators for the prognosis of diabetic wounds
Lactic acid	0–4 mM (CW-care) 0–30 mM (e-ECM)	-	Continuity: Oxygen deprivation metabolic markers; Association with inflammation severity	Concurrently assess tissue hypoxia with oxygen
Na^+^	133–146 mM	-	Imbalance: Disruption of interstitial fluid osmotic pressure → Delayed healing	Reflecting the homeostasis of the wound microenvironment
K^+^	3.2–5.7 mM	-	Imbalance: Abnormal cell membrane potential → Restoration of cell function	Reflect the metabolic state of cells
Dissolved oxygen	0–3.35 mL/L (e-ECM)	-	Low oxygen: Vascularization/Collagen deposition ↓; Macrophage recruitment is blocked High oxygen: Guided oxygen therapy intervention	Linking with ROS to evaluate oxidative stress
ROS	-	-	Upregulation-Marker of infection and chronic inflammation; Strongly associated with impaired healing	Sensitive indicators preceding clinical infection
Microbial metabolites	-	-	Specific markers: Pseudomonas aeruginosa: Pyocyanin; Staphylococcus aureus: Phospholipase A2/α-hemolysin	Pathogen typing and targeted therapy basis

**Table 2:** Comparison of Wearable Electrochemical Sensor Application Concepts.

Application Domain	Sensing Principle	Fundamental Advantages	Inherent Limitations	Clinical Adaptability
Infection Diagnosis	Specific biorecognition mechanisms (enzyme-substrate or antigen-antibody binding)	High pathogen targeting and molecular resolution capability	Bioprobe degradation and coexisting interferents	In vivo validation is needed to address complex matrix effects
Inflammation Monitoring	Immunoelectrochemical methods (labeled or label-free binding detection)	Multiplex compatibility and ultrasensitivity	Antibody instability and narrow dynamic range	Requires validation through in vivo inflammation models
Metabolite Analysis	Enzymatic catalysis or direct oxidation of electroactive species	Direct metabolic pathway mapping and real-time tracking	Enzyme denaturation and electroactive interference	Necessitates continuous in vivo monitoring
Wound Environment Tracking	Physicochemical signal conversion via electrical changes (potential/resistance/capacitance)	Wide linear range and continuous parameter monitoring	Sensor drift and mechanical stress sensitivity	Requires long-term in vivo stability verification
Therapeutic Feedback Control	Closed-loop sensing-actuation coupling triggering therapeutic delivery	Integrated diagnosis-therapy systems and adaptive intervention	System latency and limited drug payload capacity	Mandates in vivo therapeutic efficacy validation

## Data Availability

Not applicable.
